# Application of D-Amino Acids as Biofilm Dispersing Agent in Dental Unit Waterlines

**DOI:** 10.1155/2018/9413925

**Published:** 2018-01-14

**Authors:** Ruchanee Salingcarnboriboon Ampornaramveth, Nilada Akeatichod, Jesita Lertnukkhid, Nichakorn Songsang

**Affiliations:** ^1^Research Unit on Oral Microbiology and Immunology, Department of Microbiology, Faculty of Dentistry, Chulalongkorn University, Bangkok, Thailand; ^2^Pakhum Hospital, Buriram, Thailand; ^3^Trat Hospital, Trat, Thailand

## Abstract

**Aim and Purpose:**

Biofilms in dental unit waterlines (DUWLs) are extremely difficult to eliminate. Aim of this study is to evaluate the efficacy of a mixture of four D-amino acids on biofilm dispersion in DUWLs.

**Materials and Methods:**

A mixture of four D-amino acids (D-methionine, D-tryptophan, D-leucine, and D-tyrosine, 10 mM each), distilled water (control), and 0.1 M hydrochloric acid (HCl) was used in the experiment. In laboratory, pieces of DUWLs covered with biofilms were submerged in different solutions for 5 days, flushed, and measured OD^600^ of the dispersed biofilms. Remnants of biofilms on the DUWLs were evaluated by SEM. In clinic, fifteen DCUs were incubated with test and control solutions, flushed, and measured OD^600^ of the dispersed biofilms. Microbial count of DUWL output water was enumerated twice a week for four weeks.

**Results:**

There was a slight, but not significant, increase in OD^600^ of flushing water in D-amino acids group. D-amino acids effectively reduced bacterial plaque as demonstrated by SEM. Incubation with D-amino acids significantly reduced biofilms especially after the first day of flushing. Bacterial count in DUWL output water was significantly reduced after treatment with D-amino acids.

**Conclusion:**

D-amino acids are applicable as biofilm dispersing agents in DUWLs.

## 1. Introduction

Biofilms, the term for a community of microorganisms, are resistant to physical and chemical stresses. Biofilms can be found in any aquatic environment including pipelines of medical devices [1]. In dentistry, dental unit waterlines (DUWLs) consist of narrow (approximately 2–3 mm internal diameter) plastic tubes, and they deliver cooling water for dental equipment [1]. DUWLs are prone to biofilm formation which results in a heavily contaminated water output. This problem has been first identified almost 50 years ago and is still of significant concern since several reports indicate biofilm-related infection for both normal and immunocompromised patients [1, 2].

Numerous approaches have been introduced to decrease the presence of DUWL biofilms. These include both nonchemical and chemical methods. Regarding a nonchemical strategy, which includes flushing, drying, and applying an antimicrobial filter, using deionized, distilled, or even sterile water appears not to affect existing biofilms [1, 2]. The application of chemical agents has been proven to be effective [3–6]. Since regrowth of biofilms takes only a short period of time, a continuous chemical treatment is necessary. However, chemical agents can have an adverse effect on the patient's oral tissues, dental chair unit (DCU) components, as well as dental restorative materials. This is, in particular, the case for residual agents present in DUWL output water, which enter the patient's oral cavity and may even be swallowed or inhaled from aerosols generated by dental instruments [7]. Nowadays, searching a better approach for the elimination of pathogenic bacterial biofilms is still a very relevant issue in the field of infection control and prevention.

Amino acids (except glycine) can occur in two isomeric forms; these are called L- and D-forms. Only L-amino acids are produced by cells and incorporated into proteins. Therefore, most natural amino acids are in the L-form. D-amino acids are found in the cell walls of bacteria, but not in their proteins. D-amino acids have been proposed to be stress signaling and are released during depletion of nutrients to trigger the dispersion of “old” biofilms [8]. These D-amino acids help bacteria adapt to environmental challenges by modulating the structure and composition of peptidoglycans which are one of the major components in the bacterial cell wall [8–10].

D-amino acids are also involved in inhibition of bacterial biofilm formation and dispersion. Kolodkin-Gal et al. demonstrated that D-amino acids could disperse *B. subtilis* biofilm by affecting the function of amyloid fibers. When noncanonical D-amino acids incorporate into peptidoglycan, they interfere with the anchoring of amyloid fibers that normally assists in holding the biofilms together [11]. Bucher et al. reported D-leucine (D-Leu) as a noncanonical D-amino acid which competes with D-alanine (D-Ala) for the fifth pentapeptide of *B. subtilis* cell wall and interferes with transpeptidation and transglycosylation. The disturbance of the composition of the bacterial cell wall specifically interferes with biofilm formation [12]. D-amino acids including D-tyrosine (D-Tyr), D-methionine (D-Met), D-FFtophan (D-Tryp), and D-leucine (D-Leu) can prevent pellicle formation [13]. The minimal inhibitory concentration (MIC) of these D-amino acids that could inhibit biofilm formation depends on the type of D-amino acid. Testing these amino acids individually revealed that the MIC of D-Met was 2 mM, of D-Tryp was 5 mM, of D-Tyr was 3 *µ*M, and of D-Leu was 8.5 mM. D-Tyr proved to be the most effective among the four. Nonetheless, the mixture of all four amino acids is more effective than D-Tyr alone. As for the dispersion of biofilms, D-Tyr (3 *µ*M) or a mixture of D-Tyr, D-Met, D-Tryp, and D-Leu (2.5 nM each) breakdowns the biofilm pellicle of *B. subtilis* [13]. Not only this type of biofilm was affected but D-amino acids can also affect *Staphylococcus aureus* and *Pseudomonas aeruginosa* biofilms [13–16].

Recently, several studies have proposed D-amino acids as candidate molecules to be applied as a biofilm dispersing agent in endodontic treatment. Rosen et al. demonstrated that D-Leu was effective in inhibiting *E. faecalis* biofilms grown on human dentin slabs [17]. Zilm et al. demonstrated that a mixture of D-amino acids containing D-Leu, D-Met, D-Tyr, and D-Tryp significantly reduced biofilm formation of *E. faecalis*. The inhibitory effect of D-amino acids on biofilm formation was concentration dependent. The authors of this study, therefore, proposed D-amino acids as a novel biofilm inhibitor in endodontic treatment [18].

These effects of D-amino acids on biofilm dispersion together with their safety support their use as a candidate for the removal of biofilms in DUWLs. Therefore, the present study aims to investigate the efficacy of D-amino acid mixtures on the removal of biofilm in DUWLs.

## 2. Material and Methods

### 2.1. Efficacy of D-Amino Acid Mixture on Dispersing Biofilm on DUWLs In Vitro

DUWLs covered with biofilm, as was judged by eye, were obtained from in-use dental chair units (DCUs) and cut into small pieces with a length of 2.5 cm. A mixture of four D-amino acids, consisting of D-methionine (D-Met; #M9375, Sigma-Aldrich, St. Louis, MO), D-tryptophan (D-Tryp; #T9753, Sigma–Aldrich, St. Louis, MO), D-leucine (D-Leu; #L0027, TCI, Tokyo, Japan), and D-tyrosine (D-Tyr; TCI, Tokyo, Japan), was prepared by dissolving them in 0.1 M hydrochloric acid (HCl) to get a final concentration of 10 mM (each). In a preliminary set of experiments, we assessed that 10 mM was the most effective concentration of D-amino acids to be used for the removal of a biofilm.

The DUWLs were submerged in 4 mL of one of the following three solutions: (i) distilled water (negative control), (ii) D-amino acids, and (iii) 0.1 M HCl (dissolvent control). The samples were incubated at room temperature for 5 days before each sample was rinsed 3 times with 1 mL of distilled water. The dispersed biofilm from each sample was collected to sonicate and to measure the OD at 600 nm. The DUWL samples were then fixed with 2.5% glutaraldehyde for 24 hours and washed with 1 mL of PBS for 3 times. They were then dehydrated, critical-point dried, gold-sputter coated, and examined using a scanning electron microscopy (JSM-5410 LV; JEOL, Tokyo, Japan) at a magnification of ×2000. Biofilms found inside the reservoir bottle of DCUs were also incubated with test and control solutions for 5 days, flushed, and visualized with SEM.

### 2.2. Efficacy of D-Amino Acid Mixture on Dispersing Biofilm on DUWLs in a Clinical Setting

Fifteen dental chair units (DCUs) were randomly divided into 3 groups of 5 DCUs each: distilled water (negative control), D-amino acids, and 0.1 M HCl (dissolvent control). The study used DCUs of the dental school during summer break; a period during which the DCUs were not in use. Before the start of the experiment, water from the DUWLs was collected to evaluate the initial bacterial CFUs. The water samples were sonicated for 5 minutes to disperse clumps of microorganisms. Then, a serial ten-fold dilution was performed before plating a 100 *µ*L of each sample onto R2A agar plates, and these were then incubated at 35°C. The baseline CFUs were assessed after 7 days of incubation. In the D-amino acids and HCl groups, the water supplying the DCUs was replaced with a mixture of D-amino acids or 0.1 M HCl, respectively.

**Figure 1:**
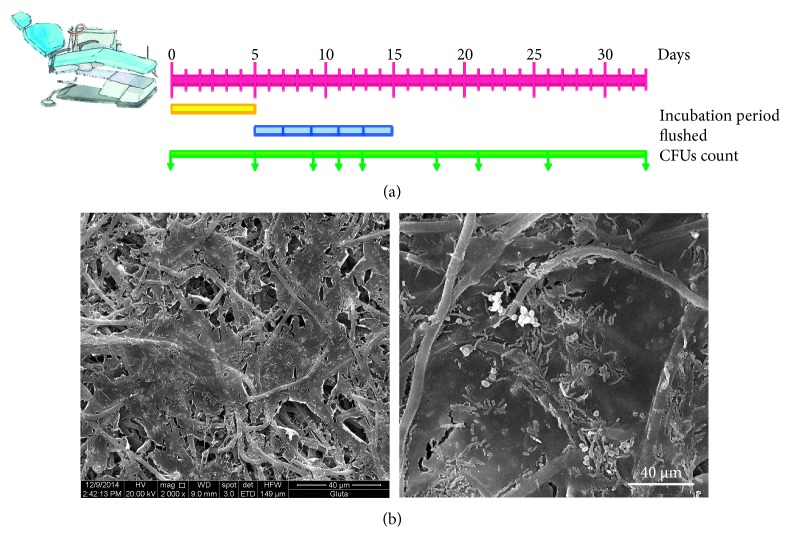
Scheme of the experimental time line (a). Incubation period: DUWLs were incubated with different solutions for 5 days (yellow bar). Flushed: DUWLs were flushed, every other day, with 1.6 L water (blue bar). Flushing water was collected to measure the dispersed biofilms by OD. CFU count: Time point at which water samples were collected for bacterial CFU counts (green bar with arrow). SEM image of biofilm mass on the inner surface of DUWLs (b). Magnification ×2000 (left image); A higher magnification image is shown on the right side.

In order to limit the amount of D-amino acid used in each DCUs, the minimum amount of water that completely flushed to the outlet of the waterline was determined by flushing diluted gentian violet into the DUWL. Fifty-five mL of the tested solution was determined as an appropriate amount to completely fill up the waterlines with testing solution. The test solutions were left in the tubing of DUWLs for 5 days. After 5 days of incubation, the waterlines were repeatedly flushed with 1.6 liters of water every other day for 10 days and the flushing water was collected to measure the OD at 600 nm. After flushing, 25 mL of water from each unit was collected and sonicated for 5 minutes. The samples were diluted ten-fold, plated on R2A agar, and incubated at 35°C for 7 days. The bacterial CFUs were then counted. Bacterial contamination in DCU output water samples was evaluated twice a week repeatedly for 4 weeks (Figure 1).

All data were analyzed using SPSS 18. The Kruskal–Wallis test and the Mann–Whitney *U* test were used for both in vitro and clinical experiments. Significance was set at *p* values < 0.05.

## 3. Results

**Figure 2:**
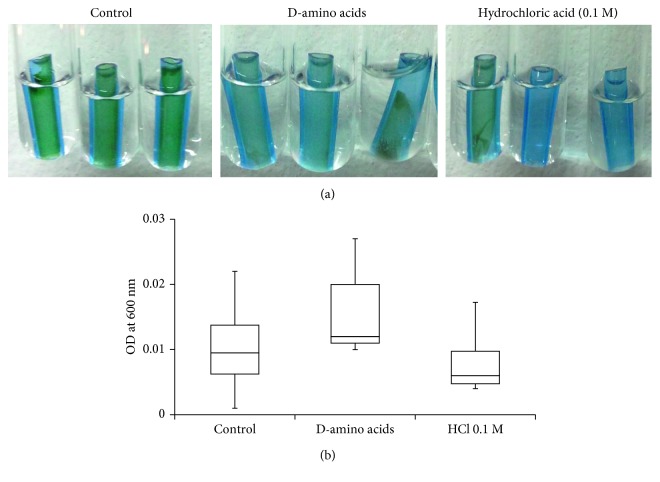
2.5 mm pieces of DUWL tube after incubation with distilled water, D-amino acids mixture (10 mM each), or 0.1 M hydrochloric acid (dissolvent control) for 5 days (a). Optical density at 600 nm (OD^600^) of dispersed biofilms in flushing water collected after the 2.5 mm pieces of DUWLs were incubated with distilled water, D-amino acids mixture (10 mM each), or 0.1 M HCl (dissolvent control) for 5 days (b).

**Figure 3:**
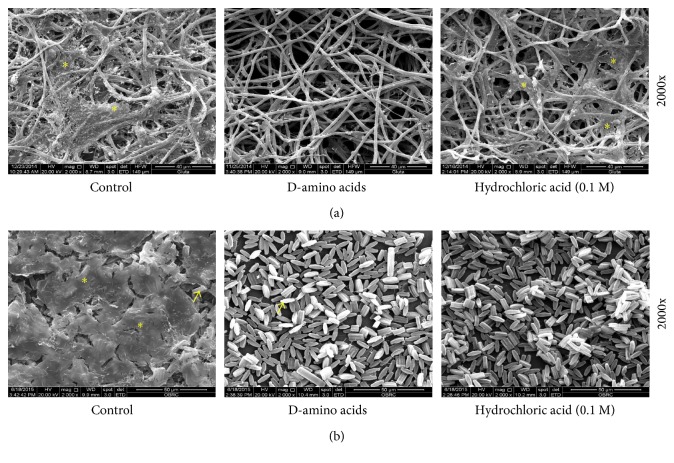
Scanning electron microscopy revealed the presence of biofilms inside DUWLs (a) and reservoir bottle of DCUs (b) after incubation with distilled water, D-amino acids mixture (10 mM each), or 0.1 M hydrochloric acid (dissolvent control) for 5 days and subsequently flushed with distilled water. Bacterial biofilm on top of the underlying structure was eliminated in the D-amino acid groups. ^∗^Bacterial biofilm. Arrow indicates surface texture of water reservoir bottle.

Biofilm mass was shown to cover the inner surface of DUWLs as shown by SEM (Figure 1). After 5 days of incubation, the biofilms on the inner surface of the tubes were seen to become loosened, some were even detached in the D-amino acids and HCl groups (Figure 2). Fragments of biofilm collected in rinsing water were used to measure OD at 600 nm. There was a trend of an increase of OD of dispersed biofilms in the D-amino acids group compared with control, though this did not prove to be statistically significant (Figure 2). Evaluation by SEM showed DUWL biofilms consisting of bacterial plaque on top of which smear-like structures were seen (Figure 3). Treatment the DUWL with D-amino acid mixtures was able to disperse the bacterial plaque that covered the biofilm (Figure 3). Biofilms dispersing effects were also found on biofilms on the inner surfaces of water reservoir bottle of the DCUs that were incubated with D-amino acids for 5 days (Figure 3).

**Figure 4:**
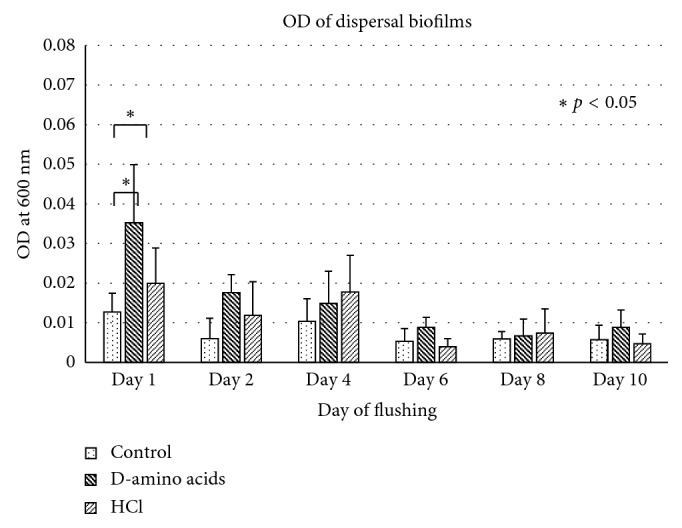
Optical density at 600 nm of dispersed biofilms in flushing water collected after the DUWLs of DCUs were incubated with distilled water (control), D-amino acids mixture (D-amino acids, 10 mM each), or 0.1 M HCl for 5 days. The DUWLs were flushed every other day for 10 days, and the flushing water was collected to measure the OD^600^ of dispersed biofilms. ^∗^Statistically different at *p* < 0.05.

**Figure 5:**
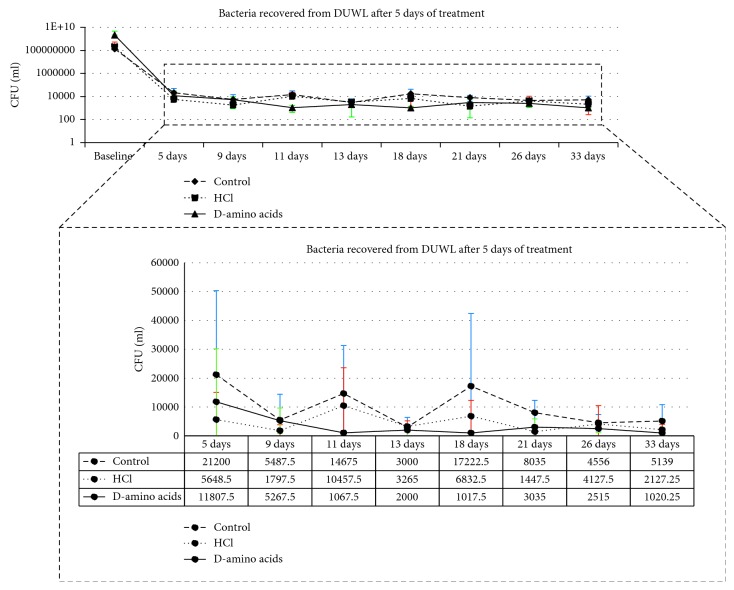
Bacterial contamination in DCU output water collected every 2-3 days for 4 weeks after the DUWLs were flushed with distilled water (control), D-amino acids mixture (D-amino acids, 10 mM each), or 0.1 M hydrochloric acid (HCl) for 5 days. A treatment of the DUWLs with D-amino acids reduced bacterial contamination in dental unit output water more than was seen in the control and HCl group.

**Figure 6:**
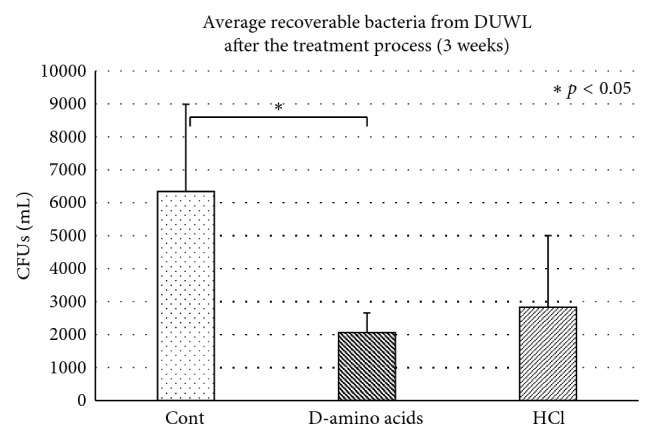
The average bacterial count in output water of the DCUs collected during the last 3 weeks after treatment of the DUWLs with distilled water (control), D-amino acids mixture (D-amino acids, 10 mM each), or 0.1 M HCl for 5 days. ^∗^Statistically different at *p* < 0.05.

In the clinical setting, the highest OD of dispersed biofilms was observed on the first day of flushing in the group treated with the D-amino acid mixture (Figure 4). After treating the DUWL with control and test solutions, bacterial contamination recovered from DCU output water was analyzed twice a week for approximately 4 weeks (Figure 5). A statistically significant lower number of average CFUs was found in the DUWL treated with D-amino acids compared with the distilled water control group (Figure 6).

## 4. Discussion

Our results demonstrated that a mixture of D-amino acids consisting of D-methionine, D-tryptophan, D-leucine, and D-tyrosine at a final concentration of each amino acid of 10 mM was effective in dispersing biofilm in DUWLs. This cleaning effect was found both in an in vitro setting and in a clinical setting. SEM analysis revealed that bacterial biofilms inside the DUWL were eliminated after incubation with the D-amino acid mixture for 5 days. Treatment of the DUWLs with the D-amino acid mixture also significantly reduced bacterial contamination in output water of DCUs. In previous studies, the efficiency of D-amino acids in dispersing biofilms has been demonstrated in vitro with single species biofilms or microbial aggregates formed by mixed cultures [13, 14, 19]. Our study is, for the first time, proposing a clinical application of a mixture of D-amino acids in the removal of biofilms in medical devices like DCUs.

Many methods both mechanical and chemical have been utilized to reduce bacterial contamination in dental unit waterlines, but none of these methods were able to disperse the existing biofilms in the complex tubing system of DUWLs. The efficiency of controlling bacterial contamination in DUWLs depends solely on chemical or physical methods of destroying or removing planktonic bacteria. Thus, the effectiveness of D-amino acid in the removal of existing biofilms in the complex dental unit tubing system appears to be an attractive way to remove biofilms from the inner parts of medical devices.

Our findings demonstrate the efficiency of D-amino acids in this process. It should be noted that also treatment of the waterlines with HCl alone partially removed biofilms. This effect might be due to the acidity of hydrochloric acid and its effect on biofilm dispersion [20, 21]. According to Tam et al., biofilm detachment of *Streptococcus mutans* occurs more rapidly as the pH drops [21]. However, HCl might corrode the metal part of the dental tubing system if it is used at a high concentration for a longer period. Although the recommended diluent for D-amino acids is 0.1 M HCl, a diluent with a neutral pH might be more suitable.

The concentration of D-amino acid mixture used in our study is slightly higher than the one used in previous studies [13, 14]. Yet, Hochbaum et al. reported a dose-dependent effect of D-amino acids with a minimum concentration of 10 mM to disperse the *Staphylococcus aureus* biofilm [14]. Kolodkin-Gal et al. proposed 3 *µ*M of D-tyrosine or a mixture of D-Tyr, D-Met, D-Tryp, and D-Leu (2.5 nM each) to disrupt the *B. subtilis* biofilm [13]. The concentrations of D-amino acids used in previous studies were effective against single species biofilms. In our study, a more complex model of naturally occurring biofilms was utilized, and therefore, a higher concentration of D-amino acids was employed.

Besides a biofilm-dispersing property, D-amino acids have also been demonstrated to inhibit or slow down the growth of biofilms [16, 22, 23]. However, there are some contradicting data regarding the efficacy of D-amino acids on inhibiting biofilm formation. Some studies demonstrated that the effect of D-amino acids was rather strain specific [15, 22, 24, 25]. One of the possible mechanisms by which D-amino acids act on biofilms was proposed to trigger the disassembly of matrix-associated amyloid fibers (see Introduction). This effect was observed in *B. subtilis* biofilms which are known to produce this type of matrix [8, 13]. However, also with organisms that do not seem to produce amyloid fibers, for example, *S. aureus* and *P. aeruginosa*, D-amino acids appear to trigger the disassembly of biofilms. The exact mechanisms involved in how D-amino acids disperse biofilms need further examination [8, 26].

## 5. Conclusion

A mixture of D-amino acids was shown to partly remove biofilms in dental unit waterlines. This study, therefore, proposes the use of such a mixture as a new approach to decontaminate bacterial biofilms in dental unit waterlines.
